# Three new species of *Rhytidhysteron* (Dothideomycetes, Ascomycota) from Mexico

**DOI:** 10.3897/mycokeys.83.68582

**Published:** 2021-09-14

**Authors:** Aurora Cobos-Villagrán, Ricardo Valenzuela, César Hernández-Rodríguez, Rosa Paulina Calvillo-Medina, Lourdes Villa-Tanaca, Luz Elena Mateo-Cid, Abigail Pérez-Valdespino, César Ramiro Martínez-González, Tania Raymundo

**Affiliations:** 1 Instituto Politécnico Nacional, Escuela Nacional de Ciencias Biológicas, Laboratorio de Micología, Prolongación de Carpio y Plan de Ayala s/n, Mexico City 11340, Mexico Instituto Politécnico Nacional Mexico City Mexico; 2 Instituto Politécnico Nacional, Escuela Nacional de Ciencias Biológicas, Laboratorio Experimental de Bacterias y Levaduras, Prolongación de Carpio y Plan de Ayala s/n, Mexico City 11340, Mexico Universidad Autónoma de Querétaro Querétaro Mexico; 3 Instituto Politécnico Nacional, Escuela Nacional de Ciencias Biológicas, Laboratorio de Ficología, Prolongación de Carpio y Plan de Ayala s/n, Mexico City 11340, Mexico Universidad Autónoma Chapingo Estado de México Mexico; 4 Instituto Politécnico Nacional, Escuela Nacional de Ciencias Biológicas, Laboratorio de Ingeniería Genética, Prolongación de Carpio y Plan de Ayala s/n, Mexico City 11340, Mexico Instituto Politécnico Nacional Mexico City Mexico; 5 Facultad de Química, Universidad Autónoma de Querétaro, Cerro de las Campanas s/n, Querétaro 76010, Mexico Universidad Autónoma de Querétaro Querétaro Mexico; 6 Universidad Autónoma Chapingo, Departamento de Fitotecnia, Instituto de Horticultura, Km 38.5 Carretera Federal México-Texcoco, Texcoco, Estado de México 56230, Mexico Universidad Autónoma Chapingo Estado de México Mexico

**Keywords:** Hysteriaceae, Hysteriales, Neotropic, phylogeny, taxonomy

## Abstract

The genus *Rhytidhysteron* is characterised by forming navicular to apothecial hysterothecia, exposing the green, yellow, orange, red, vinaceous or black colours of the hymenium which generally releases pigments in the presence of KOH. The exciple is smooth or striated, the asci bitunicate and ascospores have 1–5 transverse septa. To date, twenty-six *Rhytidhysteron* species have been described from the Tropics. The present study aims to describe three new species in the Neotropics of Mexico based on molecular methods and morphological features. Illustrations and a taxonomic key are provided for all known species of this genus. *Rhytidhysteroncozumelense* from the Isla Cozumel Biosphere Reserve, *R.esperanzae* from the Sierra Juárez, Oaxaca and *R.mesophilum* from the Sierra Madre Oriental, Hidalgo are described as new species. With the present study, the number of species of *Rhytidhysteron* known from Mexico is now increased to eight.

## Introduction

The genus *Rhytidhysteron* was described by Spegazzini (1881) and has been shown to belong to the Hysteriaceae (Boehm et al. 2009a, 2009b; Wijayawardene et al. 2020). The genus is characterised by forming hysterothecia, with lenticular or irregular, striated, or smooth openings; epithecium of various colours; excipulum composed of 1–2 layers of cells of angularis texture or globose texture. *Rhytidhysteron* presents dense hamathecium, composed of branched pseudo-paraphyses, enclosed in a gelatinous matrix; octosporic, bitunicate, cylindrical asci; 1–3 septa ascospores, constricted in the central septum, reddish-brown to brown (Spegazzini 1881; Samuels and Müller 1979; Kutorga and Hawksworth 1997; Boehm et al. 2009b; Thambugala et al. 2016).

The distribution of the genus is Pantropical. It has been reported as an endophytic fungus (Rashmi et al. 2019) and causes mycosis in humans (Chowdhary et al. 2008; Mishra et al. 2014; Mahajan et al. 2014; Chander et al. 2016).

The species with the largest distribution is *Rhytidhysteronrufulum*. It has been described from various places, with slight morphological differences depending on where it was found. *R.rufulum* have hysterothecia 1500–2000 µm long, ascospores of (19–)26–36(–43) µm and the colour of the red epithecium in Melzer’s Reagent changes to bright orange (Samuels and Müller 1979). According to Kutorga and Hawksworth (1997), the length of the hysterothecia ranges from 2500–4000 µm, ascospores from (22–)25–35(–39) µm and has dark brown to reddish epithecium in potassium hydroxide (KOH) which changes to pale greenish-brown or from red wine to intense pink. On the other hand, in the description made by Almeida et al. (2014), the size of the ascomata ranges from 800–2500 µm, ascospores from 21–32 µm and has black or red epithecium without extractable KOH pigment. The specimens from Thailand have ascomata from 900–2350 µm, ascospores from 28–36 µm and black or red epithecium are not reported to have a reaction with any reagent (Thambugala et al. 2016). Finally, Cobos-Villagrán et al. (2020), for the Mexican specimens, report ascomata of 1000–3000 µm, ascospores of 22.4–30.4 µm and orange-reddish, yellow or black epithecia changing to magenta in reaction with KOH. These morphological variations within *R.rufulum* have caused confusion in various fungal collections around the world and, as a result, they have been grouped into a complex of species (Boehm et al. 2009b; Murillo et al. 2009; Yacharoen et al. 2015; Doilom et al. 2016; Thambugala et al. 2016; Soto-Medina and Lücking 2017).

Twenty-six species are known worldwide according to the Fungorum Index (2021) and, in the last two years, it has had greater relevance, since at least seven species have been described. In the present work, morphological and molecular analyses of distinct specimens of *Rhytidhysteron* obtained from different locations in Mexico were performed. Phylogenetic relationships were inferred based on internal transcribed spacer (ITS), nuclear large subunit ribosomal DNA (LSU) and elongation factor 1-alpha (tef1). Additionally, a dichotomous key is provided with all the species described so far.

## Materials and methods

### Study zone

The specimens have been found from three different sites: one from Cozumel Island Biosphere Reserve, Quintana Roo, which is located between coordinates 20°35'20" and 20°17'16" north latitude (N) and –86°43'55" and –87°00'07" west longitude (W). The climate, according to the Köppen system, modified by García (1981), is of the AmW (I) type, warm humid with abundant rain in summer. The average annual temperature is 25.5 °C. Average annual rainfall is 1570 mm (INEGI 2013; García-Martínez et al. 2021). The type of vegetation present in the town of San Gervasio is tropical dry forest, at 0 m above sea level.

The second specimen from La Esperanza, Santiago Comaltepec, Chinantla was collected from the Sierra de Juárez in the State of Oaxaca, between coordinates 17°32' and 17°44' north latitude (N) and –96°16' and –96°36' west longitude (W); altitude between 100 and 3200 m a.s.l. La Esperanza presents different types of climates, the main ones, according to the Köppen system, modified by García (1981), are temperate humid with abundant rain in summer, C (m) and semi-warm humid with rain all year round. The temperature range is 10–26 °C. The range of precipitation is 800–4000 mm (INEGI 2008). The type of vegetation present in the town of La Esperanza is tropical cloud forest, at 1600 m a.s.l.

The last of the specimens is from Laguna de Atezca, Molango de Escamilla, which is located in the Sierra Madre Oriental in the State of Hidalgo, between the coordinates 20°42' and 20°59' of north latitude (N) and –98°41' and –98°53' of West longitude (W), altitude between 300 and 2200 m a.s.l. The Laguna de Atezca presents different types of climates, the main ones, according to the Köppen system, modified by García (1981), are semi-warm humid with rain throughout the year, ACf and temperate humid with abundant rain in summer, C (m). The average annual temperature is 17 °C. Average annual rainfall is 1438 mm (INEGI 2009). The type of vegetation present in the town of Laguna de Atezca is tropical cloud forest, at 1281 m a.s.l.

### Morphological study

The specimens were obtained by searching for dry or fallen branches in each of the localities. The material was examined following traditional techniques in mycology (Cifuentes et al. 1986). Photographs were taken using a digital camera (Nikon, D7000, Tokyo, Japan) with an 85 mm macro lens (Nikon, Tokyo, Japan). The fresh collected specimens were used to obtain morphological data such as the colour of the epithecium, growth habit and habitat. Ascomata were measured by a stereomicroscope (Zeiss 475002, Jena, Germany). Cross sections were made in the middle part of the ascomata and mounted on temporary slides in 70% alcohol and 10% KOH. Sections were observed under an optical microscope (Zeiss K-7, Jena, Germany) for the measurement of the characters of taxonomic importance.

### DNA extraction, amplification and sequencing

The DNA of each specimen of *Rhytidhysteron* spp. was obtained using the cetyltrimethylammonium bromide (CTAB) method, according to Doyle and Doyle (1987). Three molecular markers were used, the ribosomal large subunit (LSU), the internal transcribed spacer rDNA-ITS1 5.8S rDNA-ITS2 (ITS) and translation elongation translation factor 1-α (*tef1*). The primers used for LSU were LOR0f and LR5r (Vilgalys and Hester 1990), for ITS, these were ITS1f and ITS4r (White et al. 1990; Schoch et al. 2012) and *tef1* EF1-B-F1 and EF1-B-R (Wu et al. 2014). DNA amplifications were performed in a GeneAmp PCR System 9700 thermal cycler (Thermo Fisher Scientific), following recommendations by White et al. (1990) for ITS, Vilgalys and Hester (1990) for LSU and Wu et al. (2014) for *tef1*. The PCR products were verified by agarose gel electrophoresis. The gels were run for 1 h at 95 V cm^-3^ in 1.5% agarose and 1× TAE buffer (Tris Acetate-EDTA). The products were then dyed with GelRed (Biotium, USA) and viewed in a transilluminator (Infinity 300 Vilber, Loumat, Germany). Finally, the products were purified using the ExoSap Kit (Affymetrix, USA) according to the manufacturer´s instructions and were prepared for the sequencing reaction using the BigDye Terminator Cycle Sequencing Kit v. 3. 1 (Applied BioSystems). Sequencing was carried out in a genetic analyser (Sanger sequencing) by Macrogen Inc. (Seoul, Korea). The sequences of both strains of each sample were analysed, edited and assembled using BioEdit v. 1.0.5 (Hall 1999) to create consensus sequences. The consensus sequences were compared with those in the GenBank database of the National Center for Biotechnology Information (NCBI) using the BLASTN 2.2.19 tool (Zhang et al. 2000).

### Phylogenetic analyses

In order to study phylogenetic relationships, our newly produced sequences of six individuals of *Rhytidhysteron* were added to reference sequences of ITS, LSU and *tef1* (Table 1) deposited in the NCBI database (http://www.ncbi.nlm.nih.gov/genbank/). Each gene region was independently aligned using the online version of MAFFT v7 (Katoh et al. 2002, 2017; Katoh and Standley 2013). Alignments were reviewed in PhyDE (Müller et al. 2005), followed by minor manual adjustments to ensure character homology between taxa. The matrices were formed for ITS by 28 taxa (667 characters), for LSU by 31 taxa (875 characters); while the *tef1* consisted of 24 taxa (896 characters). *Gloniopsiscalami* was used as the outgroup. The aligned matrices were concatenated into a single matrix (31 taxa, 2438 characters). Five partitioning schemes were established: one for the ITS, one for the LSU, and three to represent the three codon positions of the *tef1* gene region, which were established using the option to minimize the stop codons with Mesquite v3.2 (Maddison and Maddison 2017). The best evolutionary model for alignment was sought using PartitionFinder (Lanfear et al. 2014, 2017; Frandsen et al. 2015). Phylogeny was performed with Bayesian inference using MrBayes v3.2.6 x64 (Huelsenbeck and Ronquist 2001). The information block for the matrix includes two independent runs of the MC3 chains using 10 million generations (standard deviation ≤0.1). The convergence of the chains was displayed in Tracer v1 (Rambaut et al. 2014). The highest credibility phylogram of the clades recovered with TreeAnnotator v. 1.8 (Bouckaert et al. 2014) was chosen with a 25% burn-in.

**Table 1.** Species names, strain numbers, isolation source, locality and GenBank accession numbers for the taxa used in this phylogenetic analysis. Sequences generated for this study are in bold.

Species	Isolate No.	LSU	ITS	*tef1*	Source and Locality
* Rhytidhysteron bruguierae *	MFLUCC 17–1502	MN632453.1	MN632458.1	MN635662.1	Dead stems of *Chromolaenaodorata*, Thailand
* R. bruguierae *	MFLUCC 17–1509	MN632455.1	MN632460.1	-	Dead stems of *Chromolaenaodorata*, Thailand
* R. bruguierae *	MFLUCC 17–1511	MN632454.1	MN632459.1	-	Dead stems of *Chromolaenaodorata*, Thailand
* R. bruguierae *	MFLUCC 17–1515	MN632452.1	MN632457.1	MN635661.1	Dead stems of *Chromolaenaodorata*, Thailand
R.bruguierae*	MFLU 18–0571	NG_068292.1	-	MN077056.1	Submerged branches of *Bruguiera* sp. Thailand
* R. camporesii *	KUN-HKAS 104277	MN429072.1	MN429069.1	MN442087.1	Dead stems, China
* R. chromolanae *	MFLUCC 17–1516	MN632456.1	MN632461.1	MN635663.1	Dead stems of *Chromolaenaodorata*, Thailand
*** R. cozumelense ***	**A. Cobos-Villagrán 951**	**MW9394459**	**MZ056797**	**MZ457338**	**Dead twigs of *Tabebuiarosea*, Mexico**
*** R. cozumelense ***	**T. Raymundo 7321**	**MW9394460**	**MZ056798**	**MZ457339**	**Dead twigs of *Tabebuiarosea*, Mexico**
* R. erioi *	MFLU 16–0584	MN429071.1	MN429068.1	MN442086.1	Dead stems, Thailand
*** R. esperanzae ***	**T. Raymundo 6579**	**MW9394457**	**MZ477203**	**MZ457336**	**Dead stems Mexico**
*** R. esperanzae ***	**R. Valenzuela 17206**	**MW9394458**	**MZ477204**	**MZ457337**	**Dead stems Mexico**
* R. hysterinum *	EB 0351	GU397350.1	-	GU397340.1	Dead branches, France
* R. hongheense *	KUMCC 20–0222	MW264193.1	MW264214.1	MW256815.1	Dead twigs of *Dodonaea*, China
* R. hongheense *	HKAS112348	MW541820.1	MW541824.1	MW556132.1	Dead twigs of *Dodonaea*, China
R.magnoliae*	MFLUCC 18–0719	MN989384.1	NR_170019.1	MN997309.1	Dead twigs of *Magnoliagrandiflora*, China
R.mangrovei*	MFLU 18–1894	NG_067868.1	NR_165548.1	MK450030.1	Dead twigs of mangrove, Thailand
*** R. mesophilum ***	**A. Trejo 74**	**MW9394461**	**MZ056799**	**MZ457340**	**Dead stems, México**
*** R. mesophilum ***	**A. Cobos-Villagrán 1800**	**MW939462**	**MZ056800**	**MZ457341**	**Dead stems, México**
R.mexicanum*	RV17107.1	MT626026	MT626028	-	Dead wood, Mexico
* R. mexicanum *	RV17107.2	MT626027	MT626029	-	Dead wood, Mexico
R.neorufulum*	MFLUCC 13–0216	NG_059649.1	NR_164242.1	KU510400.1	Dead wood, Thailand
* R. neorufulum *	MFLUCC 13–0221	KU377567.1	KU377562.1	-	Dead wood, Thailand
* R. neorufulum *	MFLUCC 17–2236	MH063266.1	MH062956.1	-	Dead wood, Thailand
* R. opuntiae *	GKM 1190	GQ221892.1	-	GU397341.1	Kenya
* R. rufulum *	MFLUCC 14–0577	KU377565.1	KU377560.1	KU510399.1	Woody litter, Thailand
* R. tectonae *	MFLUCC 13–0710	KU764698.1	KU144936.1	-	Dead branches, India
R.thailandicum*	MFLUCC 14–0503	NG_059648.1	NR_164241.1	KU497490.1	Dead wood, Thailand
* R. thailandicum *	MFLU 19–2373	MN989429.1	MN989428.1	MN989431.1	Dead wood, Thailand
* R. thailandicum *	MFLUCC 13–0051	MN509434.1	MN509433.1	MN509435.1	Dead wood, Thailand
Gloniopsiscalami*	MFLUCC 15–0739	NG_059715.1	KX669036.1	KX671965.1	Unknown

*Ex-type strains.

## Results

### Phylogenetic analysis

**Figure 1.**
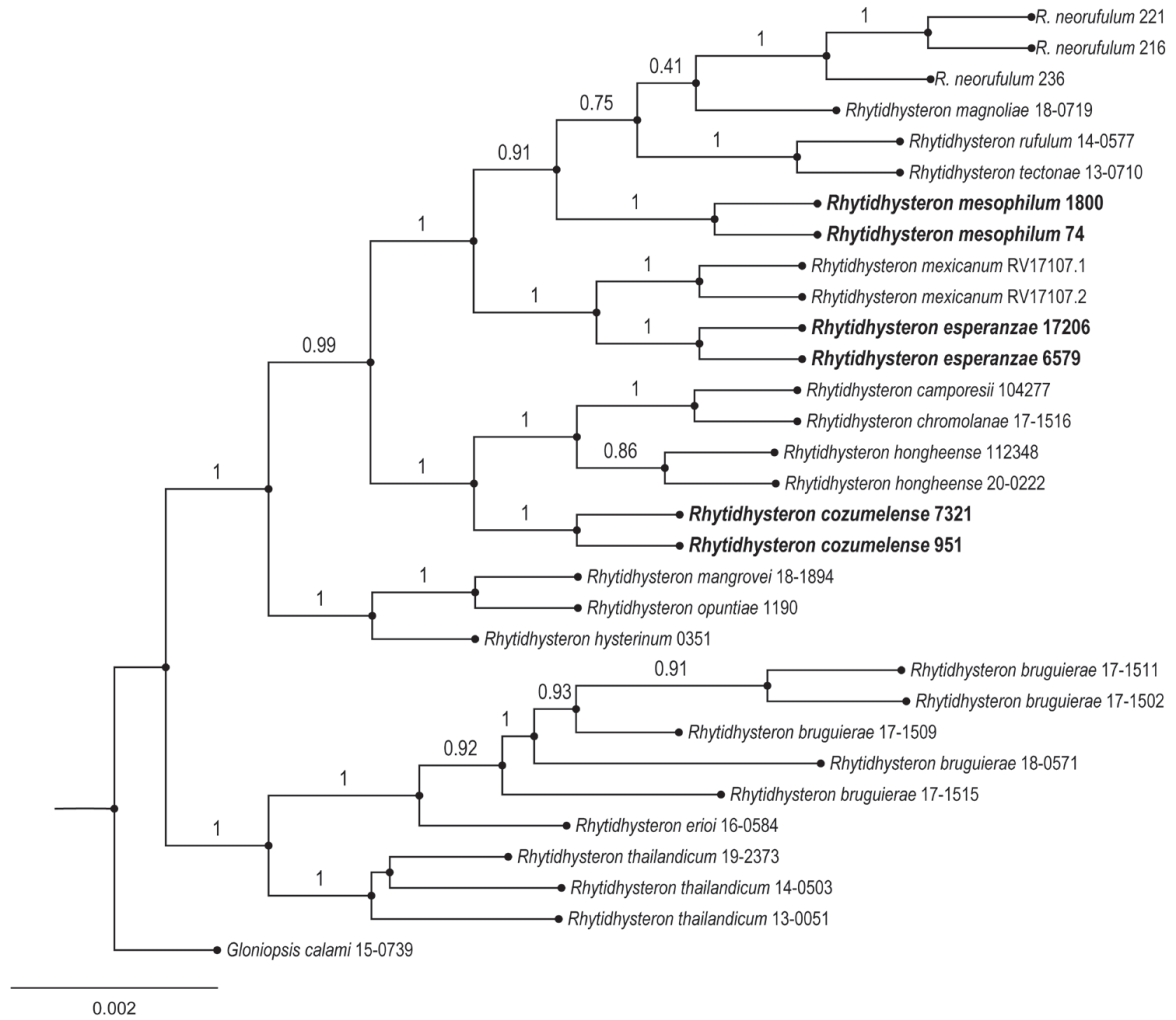
Phylogenetic relationships within the genus *Rhytidhysteron* based on a Bayesian analysis of a combined dataset of ITS, LSU and *tef1* sequence data. *Gloniopsiscalami* 150739 was used as the out-group. The posterior probabilities for each clade are shown above the branches. The new species *Rhytidhysteroncozumelense*, *Rhytidhysteronesperanzae* and *Rhytidhysteronmesophilum* are shown in bold.

The ITS, LSU and *tef1* sequences obtained from *Rhytidhysteroncozumelense*, *Rhytidhysteronesperanzae* and *Rhytidhysteronmesophilum* were deposited in GenBank (Table 1). In the Bayesian analysis, the standard deviation between the chains stabilized at 0.001 after 10 million generations, indicating that MC3 reached a stationary phase. To confirm that the sample size was sufficient, the parameter file was examined in Tracer 1.6 (Rambaut et al. 2014): all parameters had an estimated sample size of over 1,500. The posterior probabilities (PP) obtained were estimated by generating a strict consensus tree in MrBayes. Bayesian inference analysis recovered well-supported clades (PP = 1) of the three species *Rhytidhysteroncozumelense*, *Rhytidhysteronesperanzae* and *Rhytidhysteronmesophilum* (Figure 1).

### Taxonomy

#### Rhytidhysteron cozumelense

Taxon classificationFungiPatellarialesPatellariaceae

Cobos-Villagrán, R. Valenz., Hdz-Rdz., Calvillo-Medina & Raymundo sp. nov

0FE30F2C-BE51-5498-BF00-D949FFB4ECE8

839084

Fig. 2

##### Diagnosis.

Differs from *Rhytidhysteronrufulum* in its host (Bignoniaceae), size of ascomata (2.5–3.5 × 1.1–1.5 × 1.0–1.9 mm), asci (182–191 × 12–13 μm) and its reaction with KOH being faster (one to five seconds).

##### Type.

***Holotype*:** Mexico. Quintana Roo, Cozumel Municipality, San Gervasio Chen-tuk archaeological zone, 20°29'50"N, –86°50'39"W, 0 m a.s.l., 21 January 2018, A. Cobos-Villagrán 951 (ENCB), on *Tabebuiarosea* DC. (Bignoniaceae), GenBank: LSUMW9394459, ITSMZ056797, tef1MZ457338.

##### Description.

*Ascomata* hysterothecial to apothecial 2.5–3.5 mm long, 1.1–1.5 mm wide, (0.8)1.0–1.9 mm high, erumpent, solitary, boat-shaped hysterothecia, subglobose, elongated, compressed in the apex, with conspicuous longitudinal groove or cleft and becoming lenticular when mature or exposed to moisture, black, carbonaceous when dry. *Margin* involute, smooth to perpendicularly slightly striated, black. *Exciple* integrated in two layers, the first carbonaceous, glabrous, 45–100 μm thick, wide at the base, composed of pseudoparenchymal cells of *textura prismatica* (iso-radiating cells), thick-walled, the second composed of cells hyaline, thin-walled. *Pseudoparaphyses* up to 2.5 μm wide, filamentous, capitate, hyaline, septate, enclosed in a gelatinous matrix, strongly anastomosed above the asci. *Epithecium* reddish brown (8F7) when fresh, black in old specimens or when dry, becoming greyish magenta (13B5) in the presence of 10% KOH. *Asci* 182–191 × 12–13 μm, bitunicate, cylindrical, hyaline, uniseriate, octosporic, thick-walled, with a sinuous base. *Ascospores* 26–29(–31) × 9–11 (–13) μm, (x̄= 28 × 10.2 μm, n = 30), ellipsoidal to fusiform, rounded at both ends, dark brown in colour with three transverse septa, with a thick and smooth wall.

##### Distribution.

Known from a single local Island in the Cozumel Biosphere Reserve, Mexico.

##### Ecology.

Dead twigs of *Tabebuiarosea* DC. (Bignoniaceae).

##### Etymology.

The epithet refers to the Island in the Cozumel Biosphere Reserve where the species was found.

##### Specimens examined.

Mexico, Quintana Roo, Cozumel Municipality, San Gervasio Chen-tuk archaeological zone, 20°29'54"N, –86°50'43"W, 13 m a.s.l., 21 January 2018, T. Raymundo 7321, R. Valenzuela 17985 (ENCB); 17 June 2018, A. Cobos-Villagrán 1838 (ENCB).

##### Notes.

*Rhytidhysteroncozumelense* is characterised by black ascomata with a reddish brown epithecium and a smooth to slightly striated margin that, in reaction with 10% KOH, changes to greyish magenta. *R.mesophilum* has a similar reaction in KOH, but with several tones of green in the hysterothecia, a reddish orange to orange red epithecium and a perpendicularly striate with irregular slits and yellowish green pruina in margin. *R.rufulum* has a magenta reaction in KOH and strongly striated margin. *Tabebuiarosea* is reported as a new host for a *Rhytidhysteron* species.

**Figure 2.**
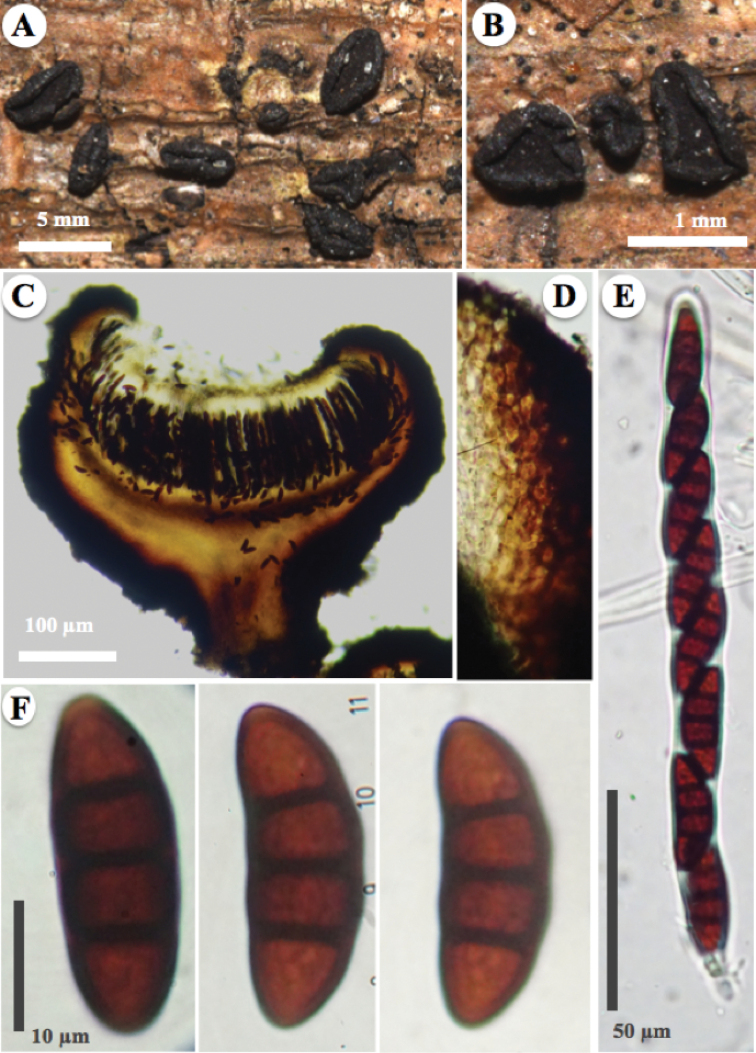
*Rhytidhysteroncozumelense* (Holotype, A. Cobos-Villagrán 951) **A** appearance of ascomata hysterothecial and apothecial on host **B** ascomata apothecial close-up, striated margin and black epithecium **C–F** microscopical features stained with alcohol (70%) and KOH (10%) reagent **C** ascomata apothecial cross-section with alcohol (70%) **D** exciple of iso-radiating cells (*textura prismatica*), close-up **E** asci **F** ascospores.

#### Rhytidhysteron esperanzae

Taxon classificationFungiPatellarialesPatellariaceae

Cobos-Villagrán, R.Valenz. & Raymundo sp. nov

D90B3B73-DC8B-5D27-A339-2DE8873D01D8

839086

Fig. 3

##### Diagnosis.

Different from most *Rhytidhysteron* species by having greyish-green ascomata with greenish-grey to yellow epithecium in the presence of KOH, and large and wide ascospores (45–47 × 17–19 μm).

##### Type.

***Holotype*:** Mexico. Oaxaca, Sierra de Juárez, Chinantla, Santiago Comaltepec Municipality, La Esperanza, Carretera Oaxaca-Tuxtepec Km 51, 17°37'55"N, –96°22'01"W, 1600 m a.s.l., 23 May 2017, T. Raymundo 6579 (ENCB). GenBank: LSUMW9394457, ITSMZ056795.

##### Etymology.

The epithet refers to the locality “La Esperanza” where the species was found.

##### Description.

*Ascomata* hysterothecial to apothecial, (2–)3–4.5 mm long, (1.2–)2–3 mm wide, (1–)1.7–2.4 mm high, superficial, solitary, rarely gregarious, boat-shaped hysterothecia, elongated, straight or flexuous, with sharp ends, opening in a discoid shape when ripe or with humidity, exposing the hymenium, taking the apothecial shape of 3–4 mm in diameter, brown (6D7), dull-green (30E4) to black. *Margin* involute, perpendicularly striate, greyish green (30C4) to dull green (30D4). *Exciple* integrated in two layers, the first carbonaceous, glabrous, 60–220 μm wide, thinning in the apical part, the middle part and the base are thicker, composed of pseudoparenchymal cells of *textura globulosa-angularis* (isodiametric cells), 11 × 10 μm, thick-walled, 3 μm wide, the second slightly pigmented to hyaline, thin-walled. *Pseudoparaphyses* up to 4 μm wide, filamentous, capitate, apical part wider, straight, hyaline, with a septum, enclosed in a gelatinous matrix, strongly anastomosed above the asci. *Epithecium* dark green (30F4) to black, becoming yellow (2A7) in the presence of 10% KOH. *Asci* (250–)265–270 × (18–)19–20 μm, bitunicate, cylindrical, rounded apex, hyaline, uniseriate, octosporic, thick-walled, with a short pedicel. *Ascospores* of (42–)45–47(–49) × (15–)17–19(–23) μm, (x̄= 45 × 17.2 μm, n = 30), ellipsoidal to spindle-shaped, rounded or pointed at both ends, reddish-brown to brown when mature, with three transverse septa, constricted at the septa, thick-walled and smooth.

**Figure 3.**
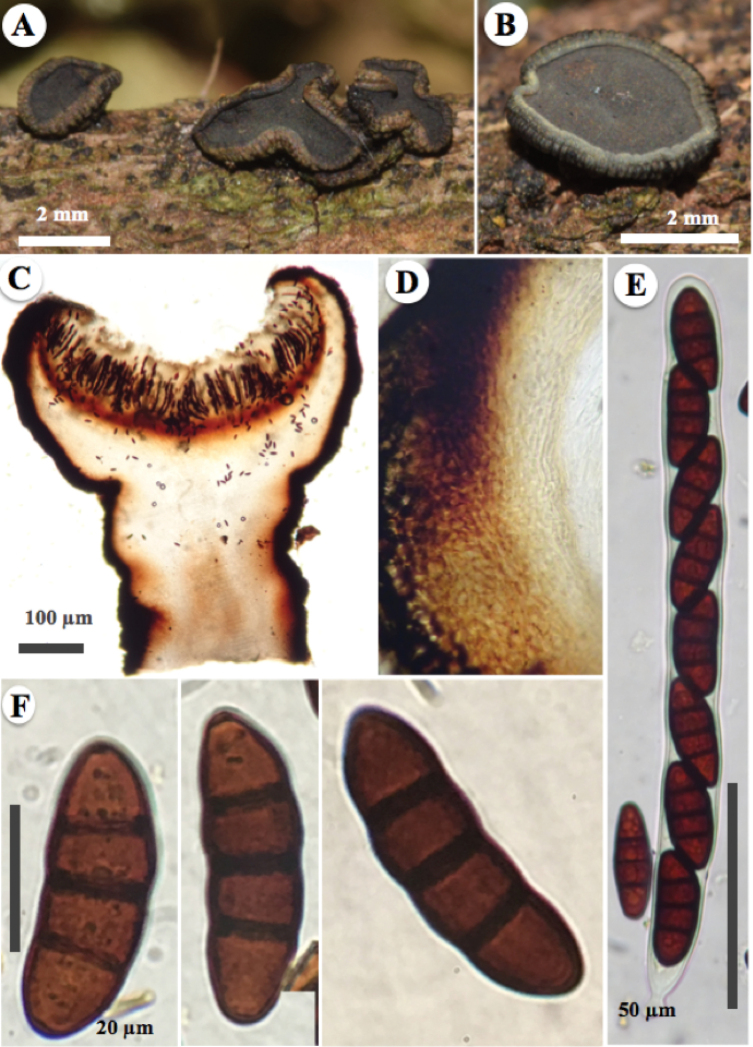
*Rhytidhysteronesperanzae* (Holotype, T. Raymundo 6579) **A** appearance of ascomata apothecial on host **B** ascomata apothecial close-up, greyish-green to dull green and striated margin and dark green to black epithecium **C–F** microscopical features stained with alcohol (70%) and KOH (10%) reagent **C** ascomata apothecial cross-section with alcohol (70%) **D** exciple of isodiametric cells (*textura globulosa-angularis*), close-up **E** asci **F** ascospores.

##### Distribution.

Known from a single locality in a forest in La Esperanza, Mexico.

##### Ecology.

Dead stems and twigs in tropical cloud forest dominated by *Oreomunneamexicana* Standl. J.-F. Leroy (Juglandaceae).

##### Specimens examined.

Mexico. Oaxaca. Sierra de Juárez, Santiago Comaltepec Municipality, La Esperanza, Carretera Oaxaca-Tuxtepec Km 51, 17°37'55"N, –96°22'01"W, 1600 m a.s.l., 22 May, 2017, R. Valenzuela 17206 (ENCB); 23 May 2017, A. Cobos-Villagrán 498 (ENCB); 25 May 2017, E. Campero 3 (ENCB), 30 April 2018, A. Cobos-Villagrán 1119 (ENCB), A. Gay AG30041814 (ENCB).

##### Notes.

*Rhytidhysteronesperanzae*, is characterised by a brown, dull-green to black exciple and dark green to black epithecium that, in reaction with 10% KOH, changes to yellow colouration. This colouration with KOH is very different than those of of *R.rufulum* and *R.neorufulum* which are magenta and violet, respectively. *R.esperanzae* have larger ascospores than *R.rufulum* (22.4–30.4 × 8–9.6 μm) and *R.mexicanum* (34–40 × 10–12 μm). Ecologically, this new species grows in a tropical cloud forest dominated by *Oreomunneamexicana* Standl. J.-F. Leroy (Juglandaceae).

#### Rhytidhysteron mesophilum

Taxon classificationFungiPatellarialesPatellariaceae

Cobos-Villagrán, R. Valenz., Hdz.-Rdz., Calvillo-Medina & Raymundo sp. nov.

DFC24A9B-FC9D-52E8-BDB9-80345652D3E4

839097

Fig. 4

##### Diagnosis.

Differs from *Rhytidhysteronrufulum* by its green-yellowish pruina on the margins, size of asci (267–282 × 15.5–16 μm) and larger ascospores (40–44 × 12–14 μm).

##### Type.

Molango de Escamilla Municipality, Laguna Atezca, 20°48'32"N, –98°44'52"W, 1281 m a.s.l., 01 June 2018, A. Trejo 74 (ENCB). GenBank: LSUMW9394461, ITSMZ056799.

##### Etymology.

The epithet refers to the type of vegetation (mountain mesophilic forest) it was collected from.

##### Description.

*Ascomata* hysterothecial to apothecial, 2.5–4 mm long, 1.0–1.5 mm wide, 1.4–1.7 mm high, superficial or erumpent, gregarious, rarely solitary, with small hysterothecial ascomata, ellipsoid to oblong and black when young, then boat-shaped hysterothecia, with some constriction in the middle part, flexuous, open in apothecioid ascomata, dark green (30F3–4), dull green (30E3–4), greyish green (30E6–7), deep green (30D-E8) to yellowish green (30B-C8) when mature, forming small ascomata within disc in old specimens. *Margin* involute, perpendicularly striate, marks are not roughness, rather irregular slits, with yellowish green (30B-C8) pruina. *Exciple* integrated in two layers, the first carbonaceous, glabrous, green yellowish, 62.5–75 μm thick, in the middle part widening more (112.5–125 μm), composed of pseudoparenchymal cells of *textura prismatica* (iso-radiating cells), the second composed of cells hyaline, thin-walled. *Pseudoparaphyses* 2.0–2.5 μm up to 3.0 μm wide, filamentous, capitate, hyaline, without septa, branched towards the apex, enclosed in a gelatinous matrix, strongly anastomosed above the asci. *Epithecium* reddish orange (7B8) to orange red (8A8), becoming greyish magenta (13D6) in the presence of 10% KOH. *Asci* 267–282 × 15.5–16 μm, bitunicate, cylindrical, hyaline, uniseriate, octosporic, thick-walled, with a sinuous base. *Ascospores* (38–)40–44(–46) × 12–14 μm, (x̄= 44.2 × 13.6, n = 30), ellipsoidal to oblong, light brown in colour, with three transverse septa, constricted at the septa, with a thick and smooth wall.

**Figure 4.**
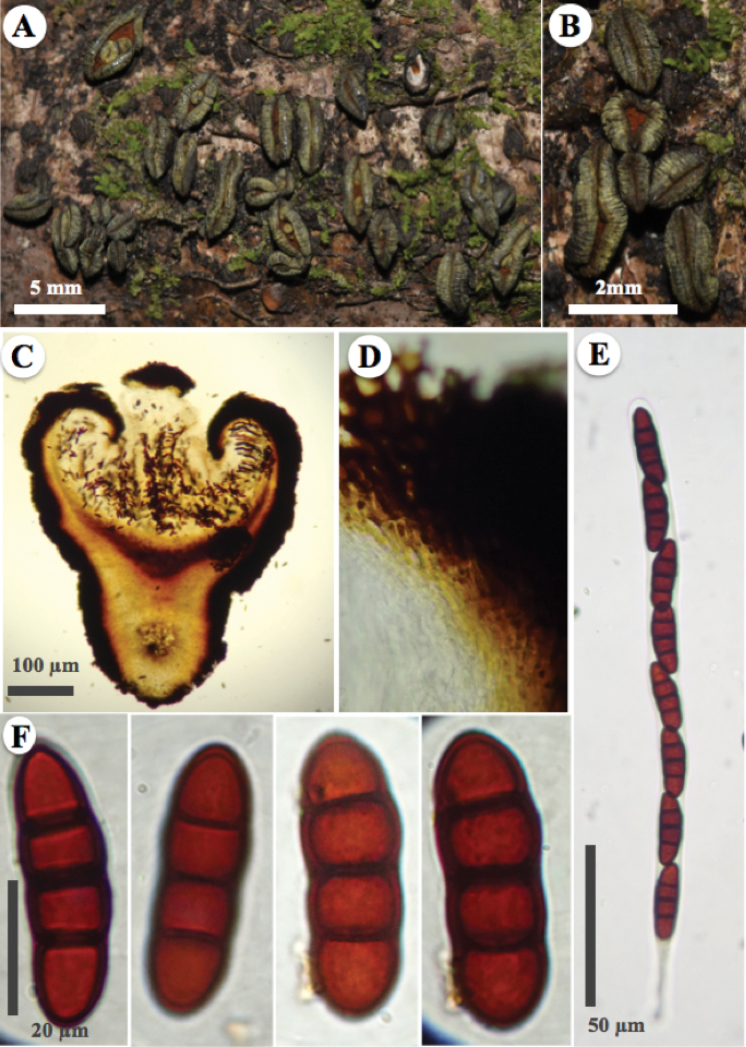
*Rhytidhysteronmesophilum* (Holotype, A. Trejo 74) **A** appearance of ascomata hysterothecial on host **B** ascomata hysterothecial close-up, striated margin with yellowish green pruina and reddish orange to orange red epithecium **C–F** microscopical features stained with alcohol (70%) and KOH (10%) reagent **C** ascomata hysterothecial cross-section with alcohol (70%) **D** exciple of iso-radiating cells (*textura prismatica*), close up **E** asci **F** ascospores.

##### Distribution.

Known from a single locality in Laguna de Atezca, Molango de Escamilla, Hidalgo, Mexico.

##### Ecology.

Dead stems in tropical cloud forest.

##### Specimens examined.

Mexico. Hidalgo, Molango de Escamilla Municipality, Laguna Atezca, 20°48'32"N, –98°44'52"W, 1281 m a.s.l., 01 June 2018; C. Herrera 40 (ENCB), A. Cobos-Villagrán 1800 (ENCB).

##### Notes.

*Rhytidhysteronmesophilum* is characterised by a dark green, dull green, greyish green, deep green to yellowish green hysterothecium, forming small ascomata within disc in old specimens. This fungus could be confused with *R.esperanzae* because both are found in tropical cloud forest (mesophilic forests) and have similar ascospores. However, *R.mesophilum* is distinguished by a reddish orange to orange red epithecium, while in *R.esperanzae*, the epithecium is dark green to black. *R.mesophilum* also resembles *R.columbiense* by the presence of a yellowish green (30B-C8) pruina in the margin, but the ascospores are larger (38–52 × 13–18 μm) and the epithecium is brown to dark brown in the second species.

A dichotomous key is presented with the species of *Rhytidhysteron* accepted by Index Fungorum (2021), including the three new species proposed in this work. The key includes the recently described *R.mexicanum* Cobos-Villagrán, Raymundo, Calvillo-Medina & R. Valenz and *R.hongheense* Wanas. It should be noted that *R.fuscum* (Ellis & Everh.) J.L. Bezerra & Kimbr. and *R.minor* (Cooke) A. Pande are not considered because the first belongs to the genus *Tryblidiella* and the second is a *nom. inval.*, because the basionym was not indicated and bibliographic reference omitted (Art. 41.5, see Art. 41.7, Melbourne).

### Key to the known species of *Rhytidhysteron*

**Table:** 

1	Ascospores submuriform	**2**
–	Ascospores transversely septate, 1–5 septa	**3**
2	Ascospores with 3–5 transverse and 1–3 longitudinal septa, 20–25 × 7.5–10 µm, epithecium brown-red, on *Cylindropuntiafulgida*; type: USA	***R.opuntiae* (J.G. Br.) M.E. Barr**
–	Ascospores with 3 transverse septa mainly and rarely with 3 transverse septa and 1 longitudinal septum, 20–33 × 9–13 µm, epithecium reddish orange, on *Dodonaeaviscosa*, type: China	***R.hongheense* Wanas.**
3	Ascospores 1–septate	**4**
–	Ascospores 3–5 septate	**5**
4	Epithecium ferruginous brown, ascospores 22–32 × 10–16 µm, on *Buxussempervirens*, *Diospyros* spp. or *Ilex* spp.; type: France	***R.hysterinum* (Dufour) Samuels & E. Müll.**
–	Epithecium orange, ascospores 24.8–29(–31) × 8.8–10(–11.2) µm, on *Acacia* spp.; type: Mexico	***R.neohysterinum* Cobos-Villagrán, Hdz.-Rdz., R. Valenz. & Raymundo**
5	Five septa in mature ascospores, 30–46 × 12–20 µm, epithecium yellowish orange, on *Pinus* spp.; type: Finland	***R.dissimile* (P. Karst.) Magnes**
–	Three septa in mature ascospores	**f**
6	Ascospores 12–15 × 5–6 µm, exciple brownish green, epithecium brown, on monocotyledonous; type: Sri Lanka	***R.beccarianum* (Ces.) Bat. & Valle**
–	Ascospores longer than 15 µm	**7**
7	Ascospores between 16 to 30 µm long	**8**
–	Ascospores longer than 30 µm	**22**
8	Ascomata with exciple and/or margin in several tones of green	**9**
–	Ascomata with exciple and margin reddish brown to black	**10**
9	Ascomata with exciple and margin vivid green, perpendicularly striate, ascospores 20–30 × 7–9 μm, constricted at the central septum, on angiosperm; type: Brazil	***R.viride* Speg.**
–	Ascomata dark brown to black with yellowish green on the margin, smooth, not striate, ascospores 23–28 × 8–11 μm, slightly constricted at the central septum, on *Chromolaenaodorata*; type: Thailand	***R.chromolaenae* Mapook & K.D. Hyde**
10	Epithecium with yellow, orange, red or green colour in some development stage	**11**
–	Epithecium brown to black in young and mature specimens	**18**
11	Epithecium yellowish green, margin perpendicularly striate, ascospores 20.3–30.4 × 7.6–10.1 μm, on *Prosopisjungiflora*; type: USA	***R.prosopidis* Peck**
–	Epithecium with yellow, orange or red colour	**12**
12	Ascomata hysterotecial, epithecium yellow, margin smooth, ascospores (19–)28–29(–31) × (8–)10–12(–13) μm constricted at the central septum, on *Tectonagrandis*; type: Thailand	***R.tectonae* Doilom & K.D. Hyde**
–	Ascomata apothecial	**13**
13	Epithecium with red tones in young or mature specimens	**14**
–	Epithecium with orange tones in young or mature specimens	**16**
14	Epithecium vivid red or cinnabar red, ascospores 19.0–24.7 × 7.6–11.4 μm, constricted at the septa, on *Quercus* sp.: type: India	***R.quercinum* (B.G. Desai & V.N. Pathak) M.P. Sharma & Rawla**
–	Epithecium dark red to black	**15**
15	Growing on mangrove tree, epithecium dark red to dark brown, ascospores 21–28 × 7.5–8.5 μm; type: Thailand	***R.mangrovei* Vin. Kumar & K.D. Hyde**
–	Growing mainly on Fabaceae, not on mangroves, epithecium orange red, red, dark red to black, 22.4–30.4 × 8–9.6 μm, type: Puerto Rico	***R.rufulum* (Spreng.) Speg.**
16	Ascospores 28–30 × 10–12 μm, on angiosperm, type: Paraguay	***R.discolor* (Speg.) Speg.**
–	Ascospores smaller than 28 μm	**17**
17	Ascospores 6.2–9 μm broad, on *Bruguiera* sp. and *Chromolaenaodorata*; type:Thailand	***R.bruguierae* Dayarathne**
–	Ascospores 9–11 μm, on angiosperm; type: Thailand	***R.erioi* Ekanayaka & K.D. Hyde**
18	Margin perpendicularly striate	**19**
–	Margin smooth to slightly striate	**20**
19	Ascospores 25–27 μm broad, on angiosperm; type: Australia	***R.scortechinii* Sacc. & Berl.**
–	Ascospores 28–30(–32) μm broad, on *Magnoliagrandiflora*; type: China	***R.magnoliae* N.I. de Silva, Lumyong S & K.D. Hyde**
20	Ascomata apothecial, ascospores 26–29(–31) × 9–11 (–13) μm, on *Tabebuiarosea* DC.; type: Mexico	***R.cozumelense* Cobos-Villagrán, R.Valenz., Hdz-Rdz., Calvillo-Medina & Raymundo**
–	Ascomata hysterotecial	**21**
21	Ascospores 25–28 × 9–11 μm, hamathecium release magenta pigment in KOH, on angiosoperm; type: China	***R.camporesii* Ekanayaka & K.D. Hyde**
–	Ascospores 20–28(-31) × 7.5–12 μm, hamathecium do not release pigment in KOH, on angiosoperm; type: Thailand	***R.thailandicum* Thambugala & K.D. Hyde**
22	Ascospores 30–40 μm	**23**
–	Ascospores longer than 40 μm	**26**
23	Margin perpendicularly striate, epithecium yellowish green to pistachio green when fresh, light green to pale green when dry, 34–40 × 10–12 μm, on angiosperm; type: Mexico	***R.mexicanum* Cobos-Villagrán, Raymundo, Calvillo-Medina & R. Valenz.**
–	Margin smooth, epithecium yellow, reddish orange or black	**24**
24	Epithecium yellow, orange to reddish orange, ascospores 27–34 × (6.5–)7–10.6 (–12.5) μm, on angiosperm; type: Thailand	***R.neorufulum* Thambugala & K.D. Hyde**
–	Epithecium black	**25**
25	Ascospores 10–12 μm broad, constricted at the central septum, on angiosperm; type: Paraguay	***R.guaraniticum* Speg.**
–	Ascospores 13–14 μm broad, constricted at the septa, on *Scutiaindica*; type: India	***R.indicum* (Anahosur) M.P. Sharma & K.S. Thind**
26	Exciple black, epithecium black, ascospores 40–45 × 15–20 μm, on angiosperm; type: Brazil	***R.brasiliense* Speg.**
–	Exciple or margin with green tones	**27**
27	Exciple and margin dark green, dull green, greyish green, deep green to yellowish green when mature, epithecium reddish orange to orange red, ascospores (38–)40–44(–46) × 12–14 μm, on angiosperm; type: Mexico	***R.mesophilum* Cobos-Villagrán, R. Valenz., Hdz.-Rdz., Calvillo-Medina & Raymundo**
–	Exciple brown, dark brown to black	**28**
28	Margin with a yellowish-green pruina, epithecium brown to dark brown, ascospores 38–52 × 13–18 μm, on angisoperm; type: Colombia	***R.columbiense* Soto-Medina & Lücking**
–	Margin greyish green to dull green, epithecium dark green (30F4) to black, ascospores (42–)45–47(–49) × (15–)17–19(–23) μm, on angiosperm; type: Mexico	***R.esperanzae* Cobos-Villagrán, R.Valenz. & Raymundo**

## Discussion

The genus *Rhytidhysteron* is a highly diverse group with a mainly Pantropical distribution (Samuels and Müller 1979). The morphological characteristics that have, so far, helped in the segregation of the species are: shape and border of the hysterothecium, ornamentation of the exciple, colour and reaction of the epithecium, and size of the ascospores which only, in some cases, have helped delimiting species, as in the case of *Rhytidhysteroncolumbiense* Soto-Medina & Lücking and *R.neohysterinum* Cobos-Villagrán, Hern-Rodr., R. Valenz. & Raymundo.

Therefore, species in which the size of spores overlap, have been clarified by molecular methods and the use of molecular markers, such as ITS, LSU, elongation factor 1 alpha (TEF1), amongst others. For example, in the case of *R.rufulum*, catalogued as a species complex based on morphology, the fungal barcodes have been helpful in describing different species that are morphologically similar (Boehm et al. 2009b; Murillo et al. 2009; Yacharoen et al. 2015; Doilom et al. 2016; Thambugala et al. 2016; Soto-Medina and Lücking 2017). In recent years, part of the taxonomy has been resolved using collections from different countries around the globe. For example, in Thailand, *R.neorufulum* and *R.thailandicum* were described in the work of Thambugala et al. 2016. In the same year, Doilom et al. (2016) described *R.tectone* on *Tectonagrandis* L. (Verbenaceae) also from Thailand.

In recent years, eight new species were described: Kumar et al. (2019) described *R.mangrovei* Vinit & K.D. Hyde, isolated from dead mangrove branches; Dayarathne et al. (2020) described *R.bruguierae* Dayarathne, also isolated from mangrove branches *Bruguiera* Lam. (Rhizophoraceae); Hyde et al. (2020) described *R.camporesii* Ekanayaka & K.D. Hyde and *R.erioi* Ekanayaka & K.D. Hyde; Mapook et al. (2020) described *R.chromolaenae* Mapook & K.D. Hyde, isolated from branches of *Chromolaenaodorata* (L.) King & Robinson (Asteraceae); Wanasinghe et al. (2021) described *R.hongheense* Wanas. isolated from dead twigs of *Dodonaea* Mill. (Sapindaceae); and in Mexico, Cobos-Villagrán et al. (2020) described *R.neohysterinum* Cobos-Villagrán, Hdez.-Rdz., R. Valenz. & Raymundo and Cobos-Villagrán et al. (2021)*R.mexicanum* Cobos-Villagrán, Raymundo, Calvillo-Medina & R. Valenz. With this new study, three more species have been described from Mexico.

In the present study, we observed that *R.cozumelense* is phylogenetically close to *R.hongheense*, *R.camporesii* and *R.chromolaenae*. The four species are similar in terms of ascospore size in the range of 23–30 × 8–13 μm and have a margin smooth to slightly striate. *R.hongheense* has slightly longer ascospores (20–33 × 9–13 µm). However, they have ascomata of contrasting sizes. *R.chromolaenae* forms smaller navicular hysterothecia, 750–885 µm diam., with orange epithecium, turning purple in KOH and is described from Chiang Rai Province, Thailand (Mapook et al. 2020). *R.camporesii* has hysterothecial ascomata of 800–1100 μm long with black epithecium that changes to magenta in KOH and it is described from Yunnan Province, China (Hyde et al. 2020). Finally, *R.hongheense* has ascomata hystherothecial 1200–2000 μm long with reddish-orange epithecium and it is described from Honghe County, Yunnan Province, China (Wanasinghe et al. 2021). *R.cozumelense* produces longer ascomata, hysterothecial to apothecial, 2500 to 3500 μm long with reddish brown to black epithecium that changes to greyish magenta in KOH and it grows on *Tabebuiarosea* DC. (Bignoniaceae).

*R.esperanzae* is phylogenetically close to *R.mexicanum*, both species described from Mexico presenting similar hysterothecial to apothecial ascomata, sizes of 2000–4500 × 1200–2500 µm and a perpendicularly striate margin. However, they differ by the colour of the ascomata and the epithecium: in *R.esperanzae*, the ascomata is brown, the exciple dull-green to black, and the epithecium dark green to black, with a yellow reaction in KOH. In contrast, in *R.mexicanum*, the exciple is completely black and the epithecium yellowish green to pistachio green when fresh, light green, pale green to lemon yellow when dry, becoming ocher to yellow gold in KOH. Another difference is the size of the ascospores which are longer and wider in *R.esperanzae*: they are (42–)45–47(–49) × (15–)17–19(–23) μm, while in *R.mexicanum*, they are 34–40(–44) × 10–12(–15) μm (Cobos-Villagrán et al. 2021).

On the other hand, *R.mesophilum* is characterised by navicular hysterothecia, striated margin with green-yellowish pruina, reddish orange to orange red epithecium that changes to greyish magenta in KOH, and long ascospores. It is related phylogenetically to *R.tectonae* and *R.rufulum*. However, it is morphologically different, including in the size and colour of the hysterothecium, colour of the epithecium, colouration in the reaction with 10% KOH and the size of asci and ascospores. The hysterothecia of *R.tectonae* are 1225–3365 µm long, with a smooth margin, yellow epithecium without reaction in KOH, ascospores (19–)28–29(–31) × (8–)10–12(–13) µm and the species grows on *Tectonagrandis* L., in Chiang Rai, Thailand (Doilom et al. 2016). In *R.rufulum*, the size of the ascomata ranges from 1500–2000 µm long, the exciple is black, the epithecium brown, orange, or reddish, changing to magenta in KOH, and the ascospores are 21–32(–39) × 8–9.6 μm (Kutorga and Hawksworth 1997; Almeida et al. 2014; Thambugala et al. 2016; Cobos-Villagrán et al. 2020). In contrast, the hysterothecia of *R.mesophilum* are 2500–4000 µm long, the epithecium orange, changing to greyish magenta in KOH, and the ascospores (38–)40–44(–46) × 12–14 μm, therefore much longer and wider.

In Mexico, the tropical dry forest is the best represented vegetation with four *Rhytidhysteron* species: *R.cozumelense*, *R.neorufulum*, *R.rufulum* and *R.neohysterinum*. This is followed by the xerophilous scrub with *R.thailandicum*, *R.rufulum* and *R.neohysterinum*, and only *R.mexicanum* in *Quercus* forest. Finally, in this study, we describe *R.esperanzae* and *R.mesophilum* in a tropical cloud forest, which is a vulnerable ecosystem and therefore these species are in danger of extinction. With the present study, the number of *Rhytidhysteron* species known from Mexico reaches a total of eight and together with Thailand, they form the countries with the most species diversity of the genus.

## Supplementary Material

XML Treatment for Rhytidhysteron cozumelense

XML Treatment for Rhytidhysteron esperanzae

XML Treatment for Rhytidhysteron mesophilum
